# From quiescence to proliferation: Cdk oscillations drive the mammalian cell cycle

**DOI:** 10.3389/fphys.2012.00413

**Published:** 2012-11-02

**Authors:** Claude Gérard, Albert Goldbeter

**Affiliations:** Faculté des Sciences, Université Libre de Bruxelles (ULB), Campus PlaineBrussels, Belgium

**Keywords:** cell cycle, computational model, Cdk oscillations, limit cycle, quiescence, proliferation, cellular rhythms

## Abstract

We recently proposed a detailed model describing the dynamics of the network of cyclin-dependent kinases (Cdks) driving the mammalian cell cycle (Gérard and Goldbeter, 2009). The model contains four modules, each centered around one cyclin/Cdk complex. Cyclin D/Cdk4–6 and cyclin E/Cdk2 promote progression in G1 and elicit the G1/S transition, respectively; cyclin A/Cdk2 ensures progression in S and the transition S/G2, while the activity of cyclin B/Cdk1 brings about the G2/M transition. This model shows that in the presence of sufficient amounts of growth factor the Cdk network is capable of temporal self-organization in the form of sustained oscillations, which correspond to the ordered, sequential activation of the various cyclin/Cdk complexes that control the successive phases of the cell cycle. The results suggest that the switch from cellular quiescence to cell proliferation corresponds to the transition from a stable steady state to sustained oscillations in the Cdk network. The transition depends on a finely tuned balance between factors that promote or hinder progression in the cell cycle. We show that the transition from quiescence to proliferation can occur in multiple ways that alter this balance. By resorting to bifurcation diagrams, we analyze the mechanism of oscillations in the Cdk network. Finally, we show that the complexity of the detailed model can be greatly reduced, without losing its key dynamical properties, by considering a skeleton model for the Cdk network. Using such a skeleton model for the mammalian cell cycle we show that positive feedback (PF) loops enhance the amplitude and the robustness of Cdk oscillations with respect to molecular noise. We compare the relative merits of the detailed and skeleton versions of the model for the Cdk network driving the mammalian cell cycle.

## Models for the CDK network driving the mammalian cell cycle

A network of cyclin-dependent kinases (Cdks) drives progression along the four successive phases G1, S (DNA replication), G2, and M (mitosis) of the mammalian cell cycle (Morgan, 1995, 2006). When cells are not in a proliferative state, they remain in a quiescent phase, denoted G0. The Cdk network driving the mammalian cell cycle is controlled by multiple regulations involving a variety of intertwined negative and positive feedback (PF) loops. Due to the complexity of this regulatory network, it is necessary to resort to computational models to obtain a comprehensive picture of the dynamics of the cell cycle. Using a detailed model previously proposed for the Cdk network driving the mammalian cell cycle (Gérard and Goldbeter, 2009), or skeleton versions of this model (Gérard and Goldbeter, 2011; Gérard et al., 2012), we will illustrate how computational models can be used to investigate the dynamics of the cell cycle.

Modeling the mammalian cell cycle is an arduous task because of the very complexity of the Cdk network. For this reason, a number of models were proposed to account for parts of the mammalian cell cycle, such as the G1 phase and the G1/S transition (Qu et al., 2003; Swat et al., 2004; Alfieri et al., 2009; Pfeuty, 2012), the restriction point in G1 (Novak and Tyson, 2004), or the G2/M transition (Aguda, 1999). A model was also proposed to account for the regulation of mammalian cell cycle progression and its gating by the circadian clock in regenerating liver (Chauhan et al., 2011). Instead of focusing on a single transition between different phases of the cell cycle, we recently proposed a model for the dynamics of the global Cdk network driving the mammalian cell cycle (Gérard and Goldbeter, 2009). This model consists of four Cdk modules, each centered around one cyclin/Cdk complex. In its most detailed form this model contains no less than 39 variables. Involving multiple negative and PF loops exerted at the levels of cyclins or Cdks, the detailed model for the cell cycle accounts for the temporal self-organization of the Cdk network in the form of sustained oscillations of the various cyclin/Cdk complexes, corresponding to the progression along the successive phases of the cell cycle (Gérard and Goldbeter, 2009). We focus below on the properties of this detailed computational model and show how it can be simplified without losing its main dynamical properties.

**Figure 1:**
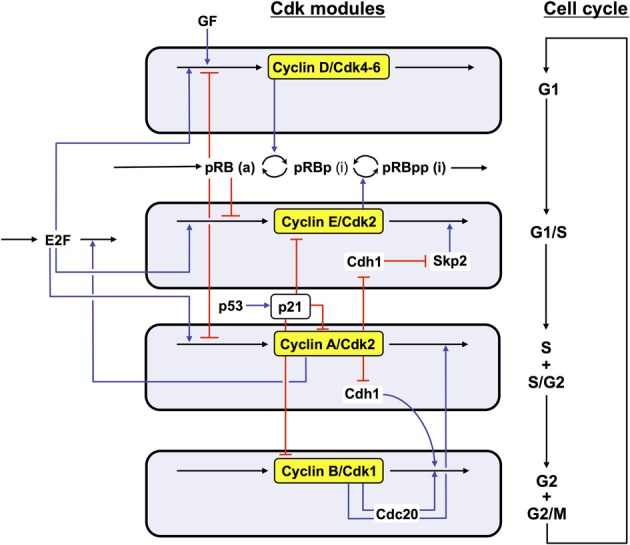
**Detailed model for the Cdk network driving the mammalian cell cycle.** The model incorporates four modules centered on the main cyclin/Cdk complexes: cyclin D/Cdk4–6, cyclin E/Cdk2, cyclin A/Cdk2, and cyclin B/Cdk1. Also considered are the effect of the growth factor GF and the roles of pRB and E2F, which exert antagonistic controls on cell cycle progression. Cyclin D/Cdk4–6 and cyclin E/Cdk2 control progression in G1 and the G1/S transition by phosphorylating, and thereby inhibiting, pRB. The rise in cyclin A/Cdk2 permits progression in S and G2, while the peak of cyclin B/Cdk1 brings about the G2/M transition. The active, unphosphorylated form of pRB inhibits E2F, which promotes cell cycle progression by inducing the synthesis of cyclins D, E, and A. The protein Cdh1, inhibited by cyclin A/Cdk2, promotes the degradation of cyclin B, and inhibits Skp2, which promotes the degradation of cyclin E; activation of cyclin A/Cdk2 thus leads to the activation of cyclin B/Cdk1 and to the inhibition of cyclin E/Cdk2. The protein Cdc20, activated by cyclin B/Cdk1, promotes the degradation of cyclin A and cyclin B, which leads to the decrease in cyclin A/Cdk2 and cyclin B/Cdk1. For the sake of clarity, not all regulations considered in the model are shown in this scheme—see supporting information in Gérard and Goldbeter (2009) for more detailed schemes of the model for the Cdk network. The combined effects of the regulatory interactions between the four Cdk modules give rise to sustained Cdk oscillations (see Figure 2) that allow the cell to progress in a repetitive, oscillatory manner along the successive phases of the cell cycle depicted on the right part of the figure (Gérard and Goldbeter, 2009).

In the detailed model for the Cdk network (see Figure 1), the synthesis of the various cyclins is regulated through the balance between the antagonistic effects exerted by the transcription factor E2F, which promotes, and the tumor suppressor pRB, which inhibits cell cycle progression. The Cdk network in turn regulates through phosphorylation the activity of E2F and pRB (Gérard and Goldbeter, 2009). Additional regulations in the detailed model for the Cdk network bear on the control exerted by the proteins Skp2, Cdh1, or Cdc20 on the degradation of cyclins E, A, and B at the G1/S or G2/M transitions, respectively. Finally, the activity of each cyclin/Cdk complex can itself be regulated through phosphorylation-dephosphorylation. The activity of cyclin D/Cdk4–6 is thus activated by phosphorylation by the cyclin-activated kinase (CAK) protein, while the Cdk2 and Cdk1 complexes are activated by the phosphatase Cdc25 and inhibited by the kinase Wee1. Multiple PF loops control the activation of the Cdks, because the phosphatases Cdc25 that activate the various Cdks are themselves activated through phosphorylation by the Cdks, while the latter inactivate their inhibitory kinase Wee1. The activity of the Cdks is further regulated through association with the protein inhibitor p21/p27 (Gérard and Goldbeter, 2009).

We previously showed that we may relinquish many of these biochemical details in building a skeleton, five-variable model for the mammalian cell cycle, without losing the key dynamical properties of the Cdk network (Gérard and Goldbeter, 2011). Thus, sustained oscillations in the various cyclin/Cdk complexes occur in the skeleton model in the presence of sufficient amounts of growth factor. Much as the detailed model, the skeleton version also accounts for the existence of a restriction point in G1 beyond which the presence of the growth factor is not needed to complete a cycle. In the first version of the skeleton model we did not incorporate regulation of Cdk1 and Cdk2 through phosphorylation-dephosphorylation, which allows for the self-amplification of Cdk activity. We extended the skeleton model by incorporating the regulation of Cdk2 and Cdk1 by the Cdc25 phosphatases and the kinase Wee1. This allowed us to assess the role of multiple PF loops on the dynamics of the Cdk network (Gérard et al., 2012).

Here, we shall first recapitulate the dynamical properties of the detailed model for the mammalian cell cycle (Gérard and Goldbeter, 2009). This model predicts temporal self-organization of the Cdk network in the form of sustained oscillations that govern the transitions between the successive phases of the cell cycle. The numerical study of the model uncovers conditions that favor the occurrence of endoreplication or tetraploidy. We will see how these results are affected by the presence of a DNA replication checkpoint, and will then turn to the coupling between the cell cycle and the circadian clock.

Dysregulation of the cell cycle is closely associated with abnormal cell proliferation. The interest of a detailed model for the mammalian cell cycle is to allow us to address the nature of the transition from quiescence to cell proliferation. We will show how the detailed model for the cell cycle can be used to illustrate the anomalous passage of cells into a proliferative regime by an alteration of the balance between factors that promote, like the transcription factor E2F, or hinder, like the tumor suppressor pRB, cell cycle progression. We will explore multiple ways that elicit the passage from quiescence to proliferation. The same factors that cause this transition may also elicit the reverse transition from proliferation to cell cycle arrest, which is often a prerequisite for cell differentiation.

The presence of multiple PF loops in the regulation of the Cdk network is associated with abrupt switches in the activation of the various cyclin/Cdk complexes that drive the transitions between the successive phases of the cell cycle. A skeleton model for the mammalian cell cycle (Gérard et al., 2012), which retains many dynamical properties of the extended model for the Cdk network, allows us to assess the effect of PF loops on the robustness of Cdk oscillations. These results illustrate the complementarity of resorting to detailed or minimal models for the molecular regulatory network that drives the mammalian cell cycle.

## Results

### A detailed model for the mammalian cell cycle

#### Oscillatory dynamics of the Cdk network

**Figure 2:**
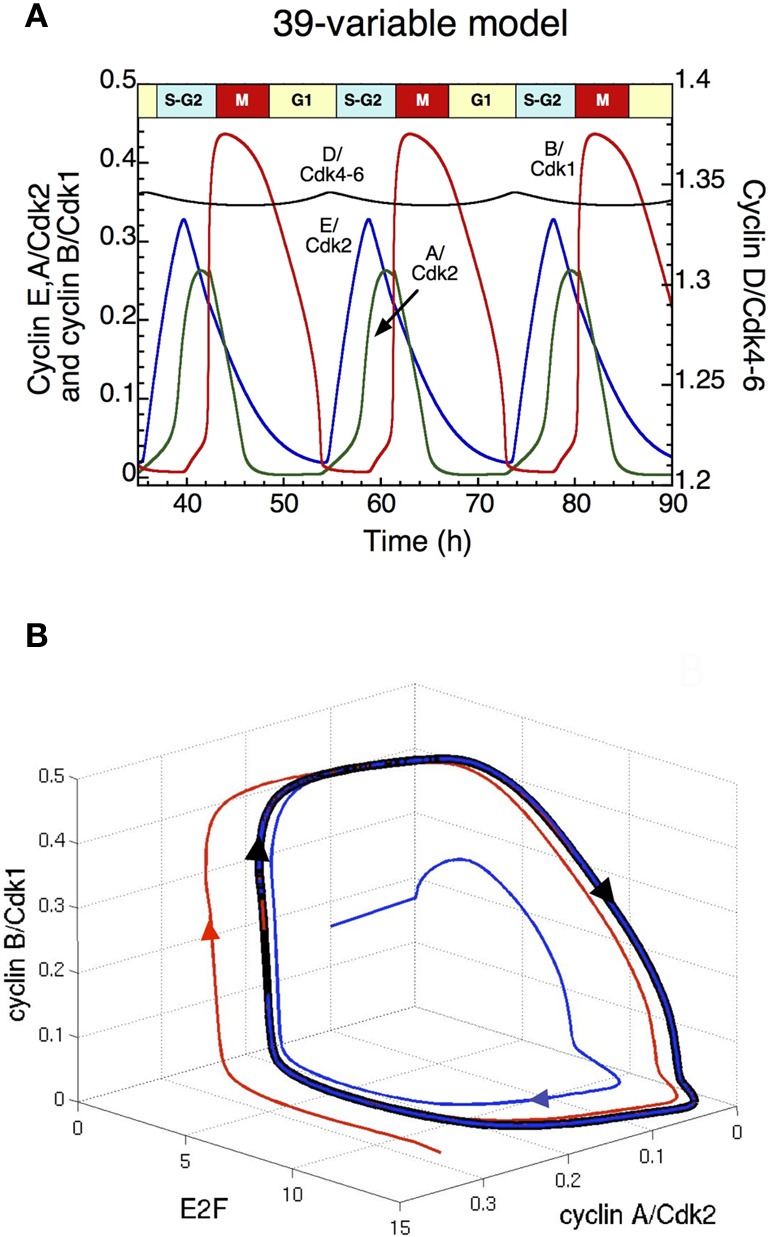
**Sustained oscillations of the Cdk network in the detailed model for the mammalian cell cycle (Gérard and Goldbeter, 2009). (A)** The time evolution of cyclin D/Cdk4–6 (in black), cyclin E/Cdk2 (in blue), cyclin A/Cdk2 (in green) and cyclin B/Cdk1 (in red) is shown in the presence of a suprathreshold level of growth factor. In the concentration space, these sustained oscillations correspond to the evolution toward a limit cycle. Cyclin D/Cdk4–6 is the total active form of the kinases, which is composed of cyclin D/Cdk4–6 and also the complex formed by cyclin D/Cdk4–6 and p21/p27 (see Gérard and Goldbeter, 2009 for more details). **(B)** Projection of the limit cycle (closed black curve) in the E2F—cyclin A/Cdk2—cyclin B/Cdk1 space. By starting from two different initial conditions (blue and red curves), the Cdk network tends to the limit cycle attractor projected in this three-dimensional space. Parameter values are as in Table S2 in Gérard and Goldbeter (2009).

The analysis of the detailed model for the Cdk network driving the mammalian cell cycle indicates that when growth factors (GF) exceed a critical value, repetitive activation of the cyclin/Cdk complexes occurs in the form of self-sustained oscillations (Gérard and Goldbeter, 2009). The ordered activation of the cyclin/Cdk complexes corresponds to the passage through the successive phases of the cell cycle. Indeed, cyclin D/Cdk4–6 and cyclin E/Cdk2 allow progression in G1 and elicit the G1/S transition, cyclin A/Cdk2 ensures progression in S and G2, while cyclin B/Cdk1 brings about the G2/M transition. Sustained Cdk oscillations in the course of time (Figure 2A) correspond, in the concentration space, to the evolution toward a *limit cycle*; this closed trajectory is unique and can be reached regardless of initial conditions (Figure 2B). In the detailed model for the mammalian cell cycle, the kinase Wee1 can inhibit Cdk1 and to a lesser extent Cdk2. Here, the initial linear increase in cyclin B/Cdk1 is due to the inactivation of Cdh1, resulting in the accumulation of cyclin B; while the subsequent abrupt peak of cyclin B/Cdk1 is due to the inactivation of Wee1 (see red curve in Figure 2A). The model indicates that the period of the cell cycle as well as the duration of the G2 phase increase with the level of Wee1. The model also predicts that high levels of Wee1 could produce endoreplication cycles, in which large-amplitude oscillations of Cdk2 are not accompanied by large-amplitude variations in Cdk1 (see section: “Endoreplication and tetraploidy”), while a further increase in the level of Wee1 generates an arrest of the cell cycle (results not shown).

#### Balance between factors that promote or hinder progression in the cell cycle

**Figure 3:**
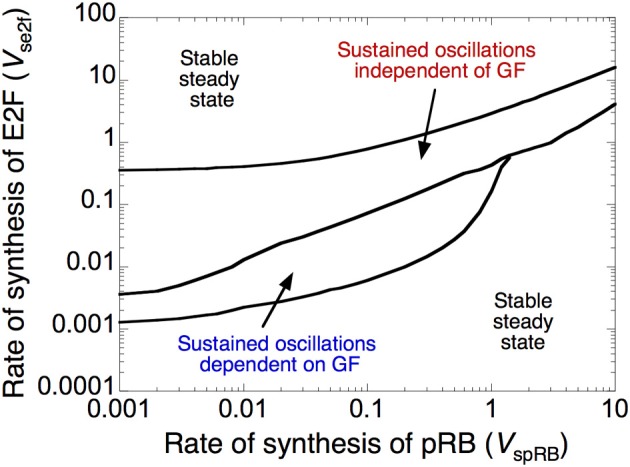
**Antagonistic effects of the transcription factor E2F and the tumor suppressor pRB on cell cycle progression.** The diagram shows the dynamical behavior of the Cdk network as a function of the rates of synthesis of E2F, *v*_se2f_, and pRB, *v*_spRB_. Regions in which the Cdk network evolves to a stable steady state, corresponding to cell cycle arrest, surround a domain of sustained oscillations, corresponding to cell proliferation. The domain of sustained oscillations is divided into two parts: in the lower part the oscillations depend on the presence of growth factor, GF (here, *GF* = 1 μM), while in the upper part of the domain they become independent of GF (*GF* = 0). Parameter values are as in Table S2 in Gérard and Goldbeter (2009).

The dynamic behavior of the Cdk network is controlled by a fine-tuned balance between factors that promote (oncogenes) or impede (tumor suppressors) progression in the cell cycle (Hanahan and Weinberg, 2000; Harbour and Dean, 2000; Chau and Wang, 2003). Here, we illustrate the antagonistic effects between two such factors, the transcription factor E2F, which promotes, and the tumor suppressor pRB, which prevents progression in the cell cycle. The dynamical behavior of the cell cycle is represented in Figure 3 as a function of the rate of synthesis of E2F, *v*_se2f_, and the rate of synthesis of pRB, *v*_spRB_. The Cdk network either evolves to a stable steady state, corresponding to cell cycle arrest and cellular quiescence, or to sustained oscillations, which correspond to cell proliferation.

The domain of sustained oscillations is divided into two sub-domains: oscillations do or do not depend on the presence of growth factor. Thus, at a given level of E2F, oscillations can occur spontaneously in the Cdk network, in the absence of GF, at sufficiently low levels of pRB, but they require the presence of suprathreshold amounts of GF when the level of pRB increases. Similarly, at a given level of pRB, Cdk oscillations cease to require the presence of GF when the rate of E2F synthesis is sufficiently large. The model therefore allows us to distinguish between two types of oscillatory behavior in the Cdk network: cell proliferation that depends on GF might correspond to normal, healthy cell proliferation, while cell proliferation independent of GF might define a “cancer-like” cell (Hanahan and Weinberg, 2000).

#### Multiple ways to trigger the transition from quiescence to cell proliferation

The detailed model for the Cdk network (Gérard and Goldbeter, 2009) is particularly useful for analyzing different ways to induce the transition between quiescence and proliferation. Such transition may result from overexpression or deletion of key proteins involved in the control of the mammalian cell cycle. Understanding the mechanisms responsible for the transition from quiescence to proliferation is of paramount importance, given that deregulation of the cell cycle as a result of overexpression of oncogenes (which activate the cell cycle) or deletion of tumor suppressors (which inhibit the cell cycle) might lead to cancer (Hanahan and Weinberg, 2000).

**Figure 4:**
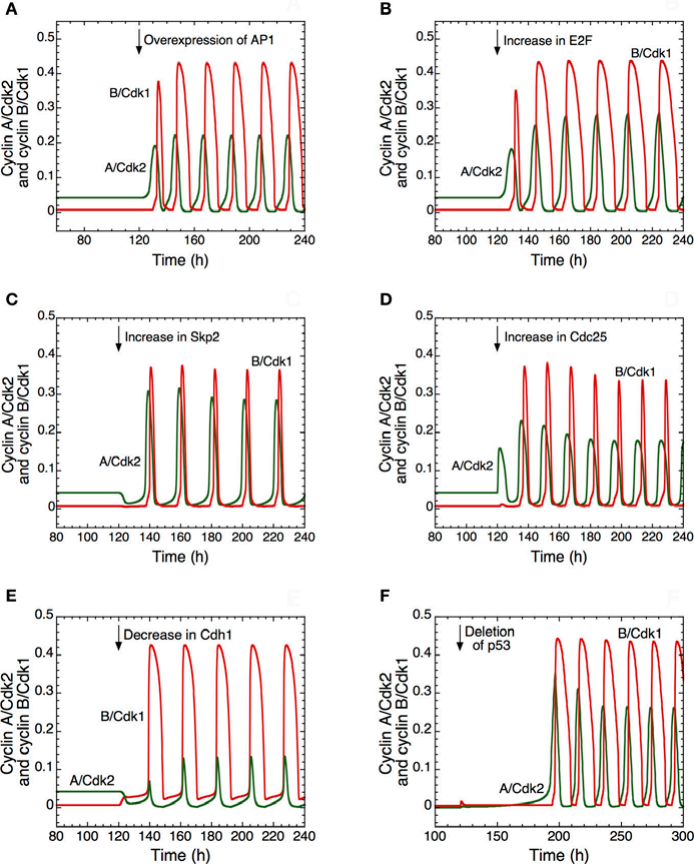
**Multiple ways to induce the passage from cellular quiescence to cell proliferation in the detailed model for the mammalian cell cycle.** The switch to sustained oscillations of cyclin A/Cdk2 (in green) and cyclin B/Cdk1 (in red) is shown following the overexpression of AP1 **(A)**, E2F **(B)**, Skp2 **(C)**, or Cdc25 **(D)**, or the decrease of Cdh1 **(E)** or p53, via a decrease in p21 **(F)**. In each case, from *t* = 120 h, the Cdk network passes from a stable steady state, corresponding to cellular quiescence, to an oscillatory regime corresponding to cell proliferation. From *t* = 120 h, the rate of synthesis of AP1, *v*_sap1_, passes from 1 to 2 μMh^−1^ in **(A)**; the rate of synthesis of E2F, *v*_se2f_, passes from 0.15 to 0.5 μMh^−1^ in **(B)**; the rate of synthesis of Skp2, *v*_sskp2_, passes from 0.15 to 0.5 μMh^−1^ in **(C)**; the rate of synthesis of the phosphatase Cdc25 acting on cyclin A/Cdk2, *v*_spai_, increases from 0.105 to 0.25 μMh^−1^ in **(D)**; the rate of synthesis of Cdh1, *v*_scdh1a_, decreases from 0.11 to 0.01 μMh^−1^ in **(E)**; the level of p53, considered as a parameter that enhances the synthesis of p21–p27, passes from 0.8 to 0 in **(F)**. For conditions **(A)** to **(E)**, the rate of synthesis of pRB, *v*_spRB_ is equal to 1.1 μMh^−1^. For condition **(F)**, the rate of synthesis of p21–p27, *v*_s1p27_ [see Equation 24 in Gérard and Goldbeter (2009)] is multiplied by (*a* + *b* · *p*53), where the first term of synthesis does not depend on p53 and the second is p53-dependent.; *v*_s1p27_ is equal to 4.8 μMh^−1^, *a* is equal to 0.2, while *b* is equal to 1. Other parameter values are as in Table S2 in Gérard and Goldbeter (2009).

Different ways to induce the transition from quiescence to proliferation are illustrated in Figure 4. In the detailed model for the Cdk network this transition is associated with the switch from a stable steady state to sustained oscillations around an unstable steady state. Thus, the model shows in **(A)** that the transition from a stable steady state, corresponding to quiescence, to a sustained oscillatory regime of the Cdk network, corresponding to cell proliferation, can be triggered by overexpression (starting in *t* = 120 h) of the protein AP1 involved in the synthesis of cyclin D that follows growth factor signaling. A similar transition can follow from overexpression of the transcription factor E2F involved in the synthesis of cyclin proteins **(B)**, overexpression of Skp2, involved in the degradation of cyclin E **(C)**, and from an increase in the level of the phosphatase Cdc25 involved in the activation of cyclin A/Cdk2 **(D)**. Similarly, the model shows that a decrease in the tumor suppressor Cdh1 involved in the degradation of cyclin B **(E)**, or a deletion of p53 **(F)**, a factor that promotes the synthesis of the Cdk inhibitor p21, also elicit the transition from quiescence to proliferation.

The model therefore suggests that abusive transitions from quiescence to proliferation may have multiple causes converging to the same effect. The switch from steady state to sustained oscillations in the Cdk network might be responsible for the tumorigenic effect of an overexpression of oncogenes such as AP1 (Smith et al., 1999) or the phosphatase Cdc25 (Parsons, 1998; Ray and Kiyokawa, 2008), of a rise in the levels of the transcription factor E2F (Gala et al., 2001) or the protein Skp2 (Yokoi et al., 2004), or a deletion of tumor suppressors such as the protein Cdh1 (Garcia-Higuera et al., 2008) or p53; the decrease in p53 is associated with a drop in the level of p21 (Nigro et al., 1989).

#### Arresting the cell cycle in G1 as a prerequisite to cell differentiation

In mammals and, more generally, in most pluricellular organisms, cells divide and proliferate in a certain undifferentiated state. When appropriate conditions are met, cells initiate a program of cell differentiation (Quaroni et al., 2000; Buttitta et al., 2007), which involves regulatory mechanisms that are not fully understood yet. However, a first step on the way to cell differentiation is to stop cell proliferation. This arrest is often achieved through overexpression of the Cdk inhibitor p21/p27 (Evers, 1999; Quaroni et al., 2000; Ilyin et al., 2003; Hara et al., 2011).

**Figure 5:**
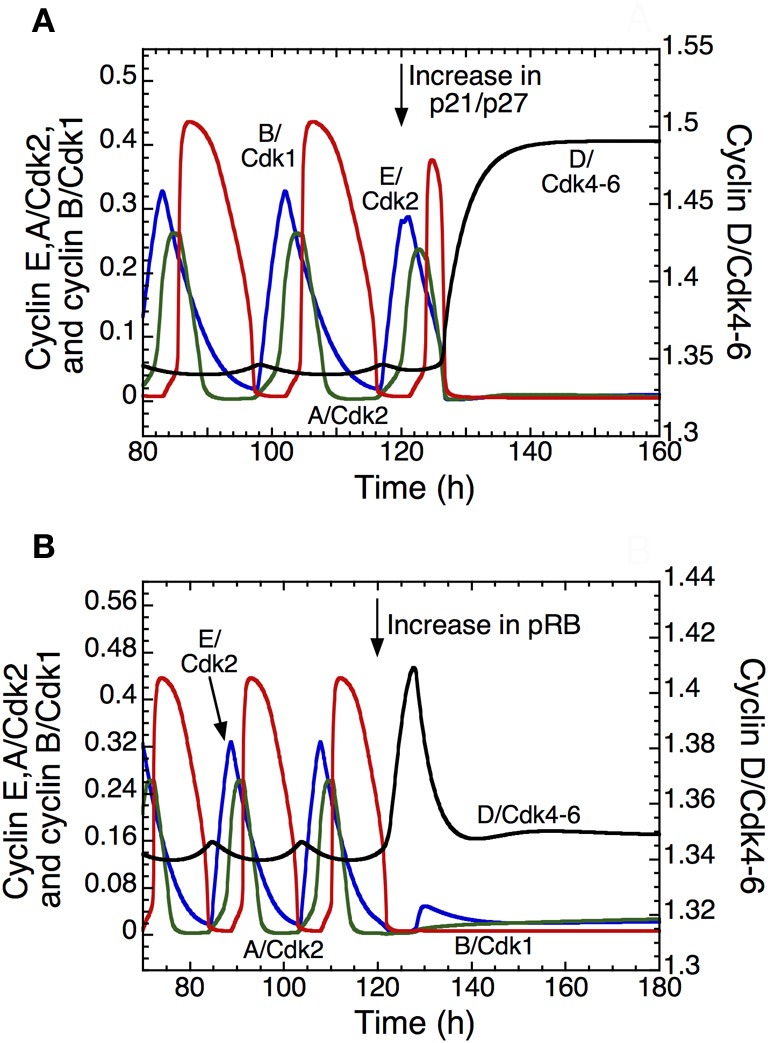
**Arresting the mammalian cell cycle in the G1 phase.** The curves show the time evolution of cyclin D/Cdk4–6 (in black), cyclin E/Cdk2 (in blue), cyclin A/Cdk2 (in green), and cyclin B/Cdk1 (in red) in the detailed model for the Cdk network. In *t* = 120 h, the transition from a sustained oscillatory regime of the Cdk network, corresponding to cell proliferation, to a stable steady state corresponding to cell cycle arrest follows the overexpression of p21–p27 when *v*_s1p27_ passes from 0.8 to 5 μMh^−1^ **(A)**, or the overexpression of pRB when *v*_spRB_ passes from 0.8 to 1.2 μMh^−1^ **(B)**. The final, stable steady state is characterized by a high level of cyclin D/Cdk4–6 and low levels of the other cyclin/Cdk complexes, which could correspond to a cell cycle arrest in G1. Other parameter values are as in Table S2 in Gérard and Goldbeter (2009).

The detailed model for the cell cycle incorporates p21 and allows us to test the effect of its increase on the dynamics of the Cdk network. Overexpression of p21/p27 (Figure 5A) or of pRB (Figure 5B) can block the progression in the cell cycle, which stops in a state characterized by a high level of cyclin D/Cdk4–6 and a low level of the other cyclin/Cdk complexes, which corresponds to the G1 phase of the cell cycle. Such an arrest of the cell cycle might represent a first step in the process of cell differentiation. Further studies are needed to clarify the coupling between cell proliferation and cell differentiation (Hara et al., 2011).

The question arises as to why the system reaches a steady state characterized by higher levels of cyclin D/Cdk4–6 and lower levels of Cdk2 and Cdk1 when p21 or pRB are overexpressed. Cyclin D/Cdk4–6 in the time series represents the total active form of the kinase Cdk4–6, which is the sum of free cyclin D/Cdk4–6 and the complex between cyclin D/Cdk4–6 and p21/p27. The Cdk inhibitor p21/p27 does not inhibit the activity of cyclin D/Cdk4–6, but inhibits the activity of Cdk1 and Cdk2. Because we assume that cyclin D/Cdk4–6 is degraded only in the free state, it is protected by its binding to p21/p27. This explains why the total level of active cyclin D/Cdk4–6 increases when p21/p27 is overexpressed, while the level of Cdk2 and Cdk1 is low (see Figure 5A). On the other hand, pRB inhibits the synthesis of cyclin D/Cdk4–6, cyclin E/Cdk2 and cyclin A/Cdk2. Thus, it is counterintuitive to reach in Figure 5B a stable steady state with a high level of cyclin D/Cdk4–6 relative to Cdk2 and Cdk1 when pRB is overexpressed. While the synthesis of cyclin E/Cdk2 and cyclin A/Cdk2 is only regulated by E2F and pRB, the synthesis of cyclin D/Cdk4–6 is also regulated by GF. Two terms are present in the synthesis of cyclin D/Cdk4–6: the first depends on GF and the second depends on pRB and E2F. In our model, the rate of synthesis that depends on GF is ten times higher than the rate of synthesis that depends on pRB and E2F. Thus, overexpression of pRB will not decrease the level of cyclin D/Cdk4–6.

#### Endoreplication and tetraploidy

**Figure 6:**
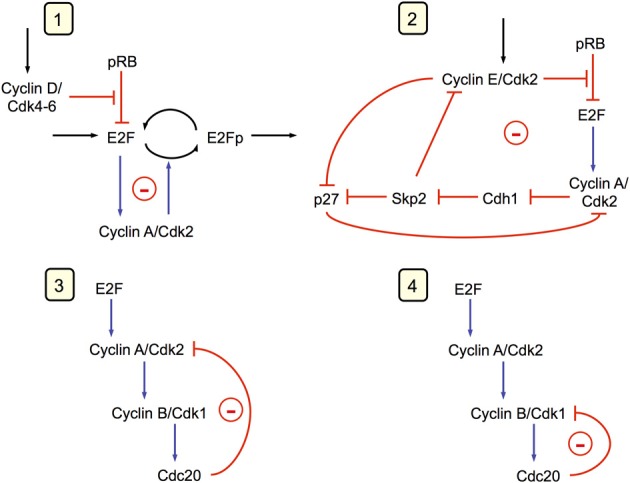
**The Cdk network driving the mammalian cell cycle contains multiple oscillatory circuits (Gérard and Goldbeter, 2009).** Four circuits containing negative feedback loops are capable of generating sustained oscillations in the model for the mammalian cell cycle. Cyclin A/Cdk2 is present in the four circuits, each of which can generate on its own sustained oscillations. In circuits **1** and **2**, oscillations can occur in the absence of cyclin B/Cdk1, which is responsible for the entry of cells into mitosis. Oscillations in Cdk2, which controls DNA replication, occur in these circuits without any peak in Cdk1, a phenomenon known as endoreplication (see Figure 7). In circuits **3** and **4**, mitotic oscillations involving repetitive activation of cyclin B/Cdk1 can occur, based on a negative feedback exerted via the protein Cdc20, which allows the degradation of either cyclin A or cyclin B. In physiological conditions, all four oscillatory circuits synchronize to produce the ordered, repetitive activation of the different modules forming the Cdk network that drives the mammalian cell cycle (see Figure 2A).

Endoreplication corresponds to uncoupling DNA replication from mitosis: the cell undergoes multiple rounds of DNA replication without entering into mitosis (Edgar and Orr-Weaver, 2001). Such a situation can also be accounted for by the model for the mammalian cell cycle. Indeed, this model predicts that the Cdk network contains at least four oscillatory circuits (see Gérard and Goldbeter, 2009, 2010 and Figure 6), which are all able to generate by themselves sustained oscillations. Two of these oscillatory circuits can produce sustained oscillations of Cdk2, which is responsible for DNA replication, without involving the kinase Cdk1 that is responsible for the occurrence of mitosis. The two other circuits involve the kinases Cdk2 and Cdk1 whose regulatory properties are at the core of the “classical” cell cycle in which each round of DNA replication is followed by entry into mitosis.

**Figure 7:**
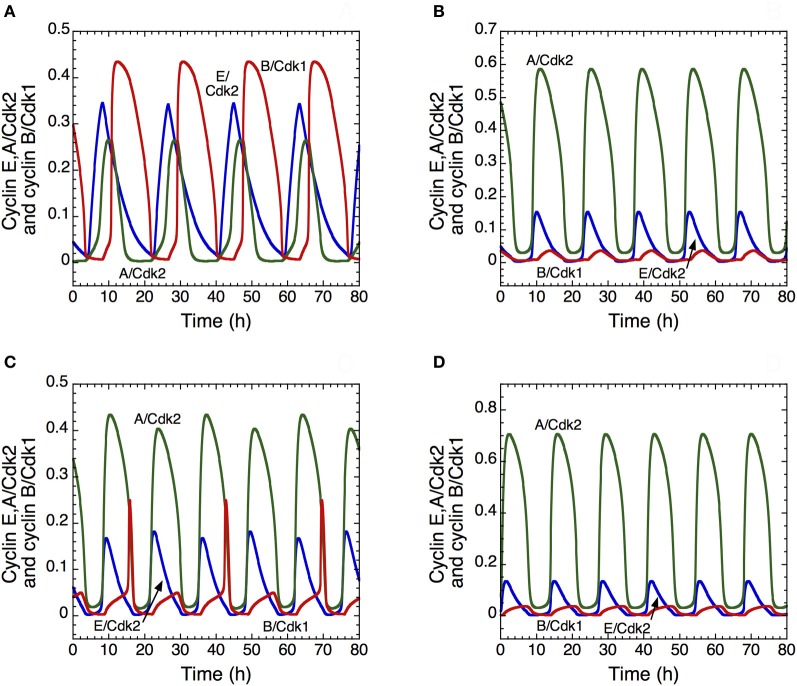
**Endoreplication or tetraploidy in the detailed model for the mammalian cell cycle.** The curves show the time evolution of cyclin E/Cdk2 (in blue), cyclin A/Cdk2 (in green) and cyclin B/Cdk1 (in red) corresponding to the “classical” mitotic cell cycle in **(A)**, to endoreplication cycles in **(B)** and **(D)**; and to tetraploidy in **(C)**. In **(B)**, the rate of inhibition of Cdh1, *V*_2cdh1_, decreases from 8 to 2.5 h^−1^, which allows the occurrence of endoreplication cycles. The rate of synthesis of cyclin B decreases from 0.05 to 0.025 μM.h^−1^ in **(C)** and to 0.02 μM.h^−1^ in **(D)**. In all cases, parameter values are as in Table S2 in Gérard and Goldbeter (2009), with the rate of synthesis of p21–p27, *v*_s1p27_, equal to 1.2 μM.h^−1^.

The model shows that, depending on their degree of interconnection, these oscillatory circuits can produce simple periodic oscillations of the Cdk network that correspond to the mitotic cell cycle, to tetraploidy, or to endoreplication. One peak of Cdk2 is generally followed by one peak of Cdk1 per cycle, which case corresponds to the progression through the “classical” mitotic cell cycle (Figure 7A). Starting from this situation, a decrease in the rate of inhibition of the protein Cdh1 that is responsible for cyclin B degradation promotes the occurrence of endoreplication cycles: sustained oscillations of Cdk2 occur without significant oscillations of Cdk1 (Figure 7B). Reducing Cdh1 inhibition leads to a decrease in the level of cyclin B; such a decrease, with similar effects, can be obtained more directly by decreasing the rate of cyclin B synthesis. Thus, from the conditions yielding the mitotic cycle in Figure 7A, a first decrease in the rate of synthesis of cyclin B, *v*_*sb*_, can elicit tetraploidy (Figure 7C), where two peaks of Cdk2 are present for one peak of Cdk1. A further decrease in *v*_*sb*_ generates again endoreplication cycles (Figure 7D). Tetraploidy will occur for the first cycle characterized by two peaks of Cdk2. For the subsequent cycles with two peaks of Cdk2, ploidy may increase if parameter conditions are maintained. Thus, for tetraploidy to occur, we must assume that a temporary change in some parameter value leads to inhibition of the Cdk1 module, and that this module may operate again when the altered parameters recover their original values.

These results suggest that the transition from a “normal” cell cycle to endoreplication or tetraploidy may be promoted in multiple ways, for example through overactivation of Cdh1 (Sorensen et al., 2000) or a decrease in cyclin B synthesis. In all cases, the model shows that the occurrence of endoreplication cycles may be readily achieved by inhibiting the activity of the Cdk1 module that controls the G2/M transition; this prediction is in agreement with experimental observations (Larkins et al., 2001).

The interactions between the different oscillatory circuits present in the Cdk network can also lead to complex dynamical behaviors such as complex periodic oscillations, quasi-periodic oscillations, and chaos (Gérard and Goldbeter, 2010). Numerical simulations based on a thorough, though limited, exploration in parameter space suggest that these complex modes of oscillatory behavior are nevertheless less frequent than the evolution toward simple periodic behavior. This conclusion holds with the view that the cell cycle is driven by simple periodic Cdk oscillations, and that such oscillations correspond to the physiological mode of dynamical behavior of the Cdk network.

#### Oscillatory dynamics in presence of DNA replication checkpoint

Checkpoints ensure that cells progress in the next phase of the cell cycle only if the preceding phase is completed correctly (Hartwell and Weinert, 1989). Checkpoints are particularly crucial in the presence of cellular damage; if DNA damage is too severe, the p53 pathway can induce apoptosis (Levine, 1997). Even during normal cell cycle progression, the correct sequence of events must be highly regulated. To illustrate how checkpoints may affect the oscillatory dynamics of the Cdk network, we incorporated into the model for the Cdk network the endogenous checkpoint that blocks cell cycle progression during DNA replication (Gérard and Goldbeter, 2009). This checkpoint is mediated by the ATR/Chk1 pathway, which inhibits the phosphatases Cdc25 that activate Cdk2 and Cdk1.

**Figure 8:**
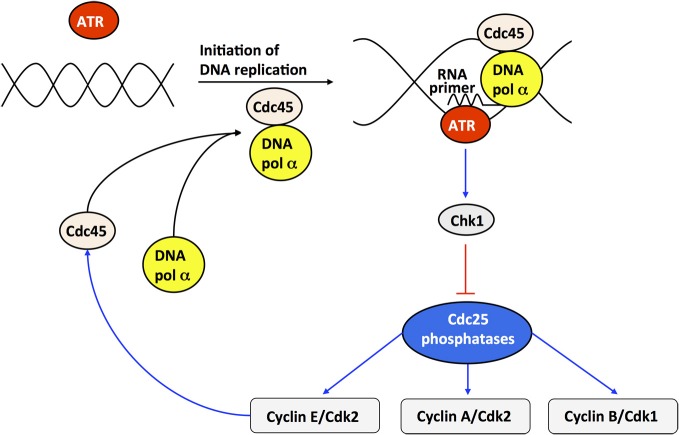
**Scheme of the DNA replication checkpoint regulated by kinases ATR and Chk1.** At the G1/S transition, cyclin E/Cdk2 activates, by phosphorylation, the anchor factor Cdc45 that allows DNA polymerase α to bind to DNA. Upon initiation of DNA replication, DNA polymerase α synthesizes an RNA primer, which binds and activates the kinase ATR. The active form of ATR activates, by phosphorylation, the kinase Chk1. Once activated, Chk1 inhibits, by phosphorylation, the phosphatases Cdc25 responsible for the activation of cyclin E/Cdk2, cyclin A/Cdk2, and cyclin B/Cdk1. The inhibition of cyclin E/Cdk2 and cyclin A/Cdk2 during DNA replication creates a checkpoint, which limits the activation of Cdc45 and thereby prevents excessive initiation of DNA synthesis at multiple points of origin of DNA replication. At the end of DNA replication, cyclin E/Cdk2 is further inhibited due to the degradation of cyclin E, brought about by the rise in Skp2, which follows from the inactivation of Cdh1 by cyclin A/Cdk2—see Figure 1 and (Gérard and Goldbeter, 2009) for further details. Cdc45 will not be active anymore, due to the inactivation of cyclin E/Cdk2, so that the activity of DNA polymerase α will decrease, and so will the concentration of RNA primer and the activity of the kinases ATR and Chk1. Because the ATR/Chk1 checkpoint promotes the inhibition of the phosphatase Cdc25 that activates cyclin B/Cdk1, the resulting inhibition of cyclin B/Cdk1 prevents cells to enter into mitosis as long as DNA replication is not completed.

At the G1/S transition, cyclin E/Cdk2 activates by phosphorylation the anchor factor Cdc45, which permits the binding of DNA polymerase α to DNA and the initiation of DNA replication (see Figure 8). The kinase ATR is activated upon binding the RNA primer synthesized by DNA polymerase α. ATR phosphorylates, and thereby activates, the kinase Chk1. Once activated, Chk1 inhibits the Cdc25 phosphatases; this inhibition blocks cell cycle progression by preventing the activation of Cdk2 and Cdk1 as long as DNA replication proceeds. Finally the decrease in Cdk2 activity, inherent to the oscillatory dynamics of the Cdk network, inhibits DNA polymerase at the end of the S phase (Dart et al., 2004). The subsequent inhibition of ATR and Chk1 relaxes the inhibition of the phosphatases Cdc25 and thereby permits the rise in the activity of cyclin B/Cdk1 that will elicit the G2/M transition [see Figure 8 and (Gérard and Goldbeter, 2009) for further details about the molecular regulatory mechanisms of the DNA replication checkpoint]. Incorporation of the DNA checkpoint involves the addition of five new variables, so that the extended, detailed model now counts 44 variables instead of 39.

**Figure 9:**
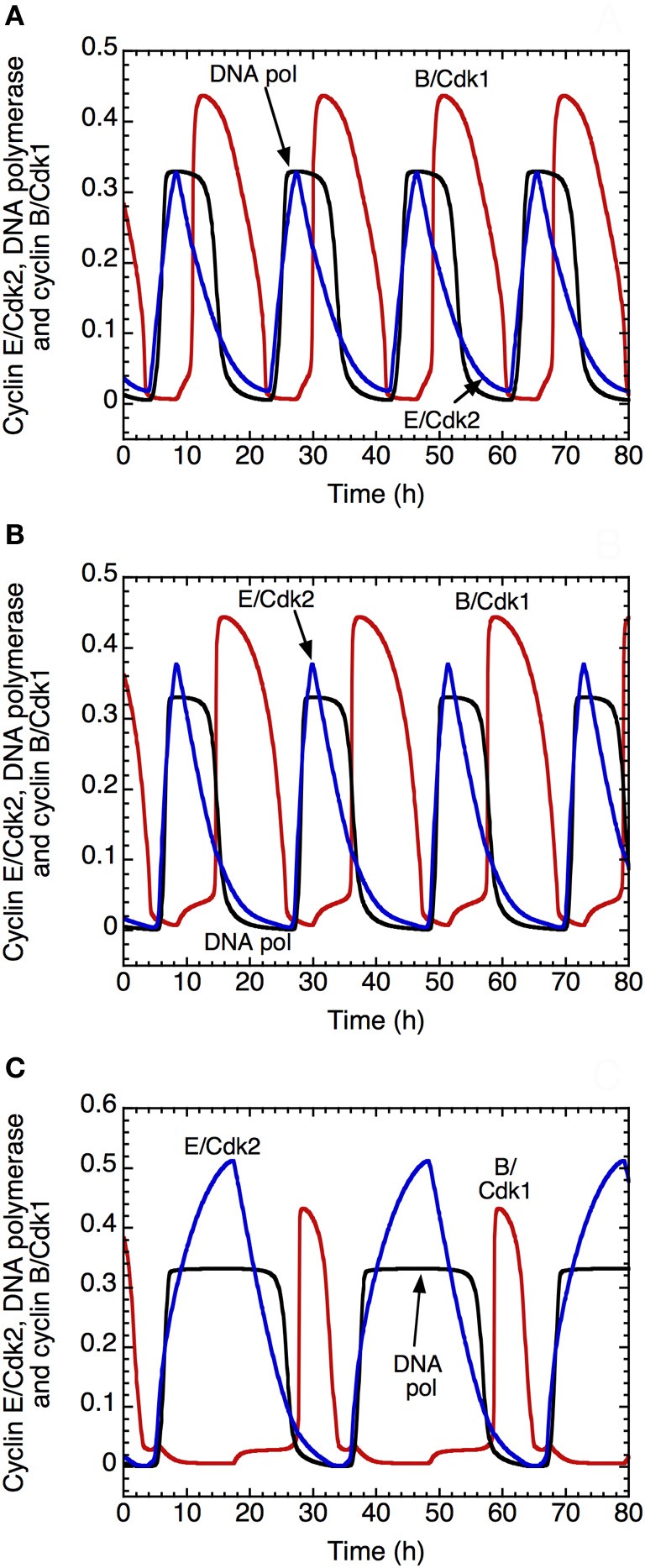
**Effect of the DNA replication checkpoint on Cdk oscillations in the detailed model for the Cdk network driving the mammalian cell cycle.** The time evolution of cyclin E/Cdk2 (in blue), DNA polymerase α (in black) and cyclin B/Cdk1 (in red) is shown **(A)** in the absence of activation of the kinase ATR driving the DNA replication checkpoint, with *k*_aatr_ = 0, or in the presence of low **(B)** or strong activation **(C)** of the kinase ATR, with *k*_aatr_ = 0.01 μM^−1^.h^−1^ in **(B)** and 0.024 μM^−1^.h^−1^ in **(C)**. The presence of the DNA replication checkpoint slows down the progression in the cell cycle: the period of the cell cycle clock passes from 19 h in **(A)**, to 21.6 h in **(B)** and 31 h in **(C)**. Moreover, the DNA replication checkpoint improves the separation between the phase of DNA replication, represented by the peak of DNA polymerase α and the phase of mitosis, represented by the peak of cyclin B/Cdk1. Other parameter values are as in Table S2 in Gérard and Goldbeter (2009).

The model shows that the DNA replication checkpoint does not alter qualitatively the oscillatory nature of the dynamics of the Cdk network. However, it slows down the progression in the cell cycle and allows for a better separation between DNA replication and mitosis (Figure 9). Indeed, when the checkpoint is inactivated, we observe an overlap between the peak of activity of DNA polymerase α, which is a marker of the S phase, and the peak of cyclin B/Cdk1, which corresponds to the M phase (Figure 9A). In the presence of mild (Figure 9B) or strong activation (Figure 9C) of the checkpoint, the progression in the cell cycle slows down: the period of the cell cycle passes from 19 h in **(A)** to 21.6 h in **(B)** and 31 h in **(C)**. Moreover, the checkpoint allows for better separation between DNA replication and mitosis (compare panel C with panels B and, even more strikingly, A). This ensures that the cell completes DNA replication before entering mitosis.

#### Mechanism of oscillations in the Cdk network

The dynamics of the Cdk network is governed by multiple positive and negative feedback loops that involve the various cyclin/Cdk complexes. At the end of the network, the last Cdk module that controls the G2/M transition is regulated by a negative feedback loop between cyclin B/Cdk1 and APC/Cdc20 (see Figure 1). The earlier modules that control the progression from G1 to S, G2, and M harbor a cascade of bistable switches in the activation of the various cyclin/Cdk complexes. Indeed, as previously stressed in experimental as well as theoretical studies, PF loops control the dynamics of the G1/S as well as the G2/M transitions of the cell cycle (Hoffmann et al., 1994; Pomerening et al., 2003; Sha et al., 2003; Chen et al., 2004; Yao et al., 2008). PF is either direct, as in the activation by cyclin A/Cdk2 and cyclin B/Cdk1 of their activating Cdc25 phosphatase, or indirect, as in the inhibition by cyclin B/Cdk1 of its inhibitory kinase Wee1.

**Figure 10:**
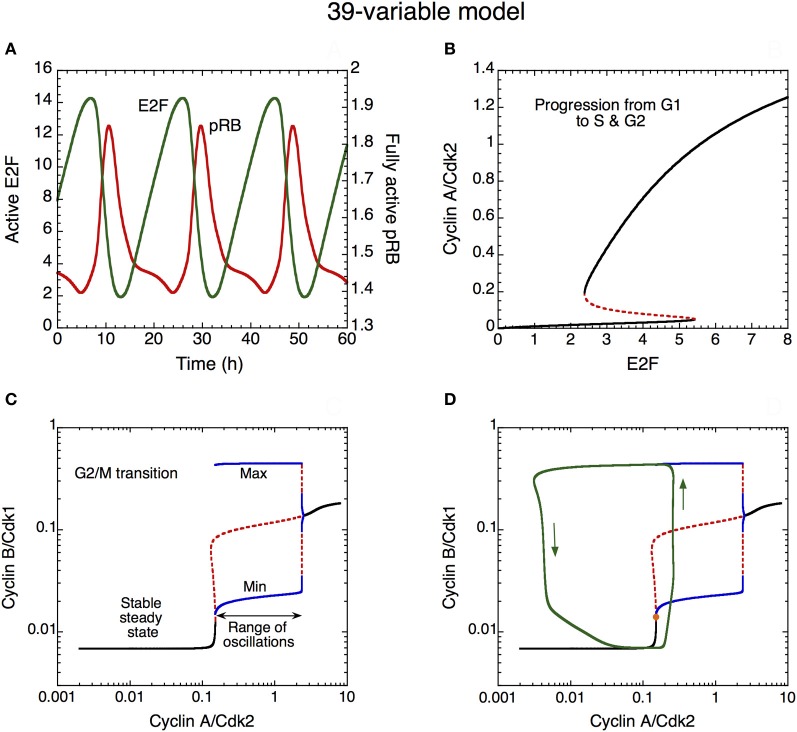
**Mechanism of oscillations in the Cdk network. (A)** The curves show the time evolution of the active, unphosphorylated, forms of E2F (in green) and pRB (in red) which oscillate in antiphase. **(B)** Bifurcation diagram showing the steady-state level of cyclin A/Cdk2 as a function of E2F, considered as a parameter. Two stable steady states coexist in the domain of bistability. The sharp increase in cyclin A/Cdk2 above a critical level of E2F favors progression from the cell cycle phase G1 to S and G2. **(C)** Bifurcation diagram showing the steady-state level of cyclin B/Cdk1 as a function of cyclin A/Cdk2, considered as a parameter. A domain of sustained oscillations exists above a critical level of cyclin A/Cdk2; the upper and lower blue curves denote the maximum and minimum of cyclin B/Cdk1 oscillations as a function of cyclin A/Cdk2. The rise in the level of cyclin A/Cdk2 pushes transiently the system into the domain of sustained oscillations, which permits the activation of cyclin B/Cdk1 at the G2/M transition. The rise in cyclin B/Cdk1 leads to the activation of the protein Cdc20, which promotes the degradation of cyclins A and B; this creates a negative feedback loop in the activation of cyclin A/Cdk2 and cyclin B/Cdk1. The subsequent decrease in the levels of cyclin A/Cdk2 and cyclin B/Cdk1 resets the system and a new cell cycle starts again in G1 if GF is present in sufficient amount. Black curves in **(C)** represent stable steady states, red dashed curves represent unstable states. **(D)** Superimposed on the bifurcation diagram of cyclin B/Cdk1 *vs.* cyclin A/Cdk2 in **(C)** is the projection of the limit cycle oscillations (green trajectory, with arrows indicating the direction of movement along the limit cycle) in the plane defined by cyclin A/Cdk2 and cyclin B/Cdk1 (Gérard and Goldbeter, 2009). Using E2F as a parameter, the bifurcation diagram in **(B)** was established by means of the program AUTO (Doedel, 1981) applied to the kinetic equations of the cyclin A/Cdk2 module, i.e., Equations 20–22, 28 and 29 (see Gérard and Goldbeter, 2009) from which the terms related to p21–p27, cyclin E/Cdk2 and pRB were removed, so as to keep this module isolated from the other modules. Using cyclin A/Cdk2 as a parameter, the bifurcation diagram in **(C)** and **(D)** was established by considering the kinetic equations of the cyclin B/Cdk1 module, i.e., Equations 26–32 and 34–39 (see Gérard and Goldbeter, 2009) from which the terms related to p21–p27 were removed, so as to keep this module isolated from the other modules.

The dynamics of the Cdk network that governs progression from G1 to S, G2, and M is illustrated in Figure 10 in the detailed model for the Cdk network driving the mammalian cell cycle. First the time evolution of pRB and E2F is illustrated in **(A)**. These antagonistic factors oscillate in antiphase. A high level of pRB and a low level of E2F characterize the G1 phase of the cell cycle. By eliciting the synthesis of the various cyclins, the rise in E2F promotes progression in the G1, S, and G2 phases of the cell cycle.

A bifurcation diagram illustrating the dynamical behavior of cyclin A/Cdk2 as a function of the transcription factor E2F (considered as a parameter) is shown in **(B)**. This second module of the network controls the progression from G1 to S and G2. In early G1, the level of E2F is low, which allows the maintenance of a low level of cyclin A/Cdk2. The rise in E2F during G1 elicits an abrupt increase in the level of cyclin A/Cdk2, as soon as E2F reaches the limit point of the bistable switch. Such an abrupt activation elicits progression in S and G2 and contributes to render the G1/S transition irreversible **(B)**. The bistable behavior in the activation of Cdk2 results from the presence of the PF regulation of cyclin E/Cdk2 (not shown) and cyclin A/Cdk2, via the activation of their phosphatase Cdc25. Later, during G2, the level of E2F will decrease because cyclin A/Cdk2 promotes its degradation by phosphorylation (Gérard and Goldbeter, 2009; see also Figure 1). This regulation provides yet another illustration of the principles of cell cycle control: each module in the Cdk network activates the subsequent modules and inhibits the previous modules in the network.

The abrupt rise in the level of cyclin A/Cdk2 permits the activation of cyclin B/Cdk1 at the G2/M transition: the increase in cyclin A/Cdk2 pushes the Cdk network into a domain of sustained oscillations of Cdk1 (see the bifurcation diagram established in Figure 10C for cyclin B/Cdk1 as a function of cyclin A/Cdk2, considered as a parameter). The occurrence of oscillations results from the negative feedback loop between cyclin B/Cdk1 and Cdc20 (see Figure 1). However, the entry of Cdk1 in the domain of sustained oscillations in **(C)** is only transient. This is made clear in **(D)** where the projection of the limit cycle trajectory followed by the full Cdk network in the cyclin A/Cdk2 *vs.* cyclin B/Cdk1 plane is superimposed on the bifurcation diagram shown in **(C).** There is only one peak of Cdk1 when the last module enters the oscillatory domain: indeed, soon after this peak, cyclin A/Cdk2 begins to decrease because cyclin B/Cdk1 activates, through phosphorylation, the protein Cdc20 that triggers degradation of both cyclins A and B. As cyclin A/Cdk2 decreases, soon followed by a decrease in Cdk1, the Cdk1 module leaves its domain of sustained oscillations (**C** and **D**). This completes the M phase of the cell cycle and leads to a return to G1, characterized by low levels of activity of the various cyclin/Cdk complexes.

The detailed model for the Cdk network thus shows that the presence of multiple PF loops elicit a cascade of abrupt activation of the various cyclin/Cdk complexes, which controls the progression from G1 to S, G2, and M. At the G2/M transition, the cell enters transiently into an oscillatory regime; the pulsatile increase in Cdk1 at the same time triggers the M phase and resets the cycle. The cell division cycle can thus be viewed as a cascade of dominoes, controlled by PF loops, which drives the ordered transition along the successive phases of the cell cycle. The negative feedback loops between Cdk1 and Cdc20 at the end of the network turns the bistable behavior of the different Cdk modules into a global limit cycle oscillator. The reset brought about by the rise in Cdk1 allows the cell to start a new cycle spontaneously if appropriate conditions are met. The mammalian cell cycle thus behaves as a self-organized, oscillating cascade of dominoes. Such a conclusion reconciles two views of the cell cycle, as dominoes and clock (Murray and Kirschner, 1989).

#### Coupling the cell cycle to the circadian clock

Several molecular components of the cell cycle network are regulated in a circadian manner (Fu et al., 2002; Matsuo et al., 2003; Gréchez-Cassiau et al., 2008). In the Cdk network that governs progression in the cell cycle, the synthesis of the kinase Wee1, which inhibits the G2/M transition, is enhanced by the complex CLOCK/BMAL1 that plays a major role in the circadian clock network (Matsuo et al., 2003). Another component of the circadian network, REV-ERBα, inhibits the synthesis of the Cdk inhibitor p21 (Gréchez-Cassiau et al., 2008). Moreover, the synthesis of the oncogene c-Myc, which promotes G1 cyclin synthesis, is repressed by CLOCK/BMAL1 (Fu et al., 2002). By coupling the detailed model for the cell cycle (Gérard and Goldbeter, 2009) to a model for the circadian clock in mammals (Leloup and Goldbeter, 2003), we investigated the conditions in which the cell cycle can be entrained through these various modes of coupling to the circadian clock (Gérard and Goldbeter, 2012a).

**Figure 11:**
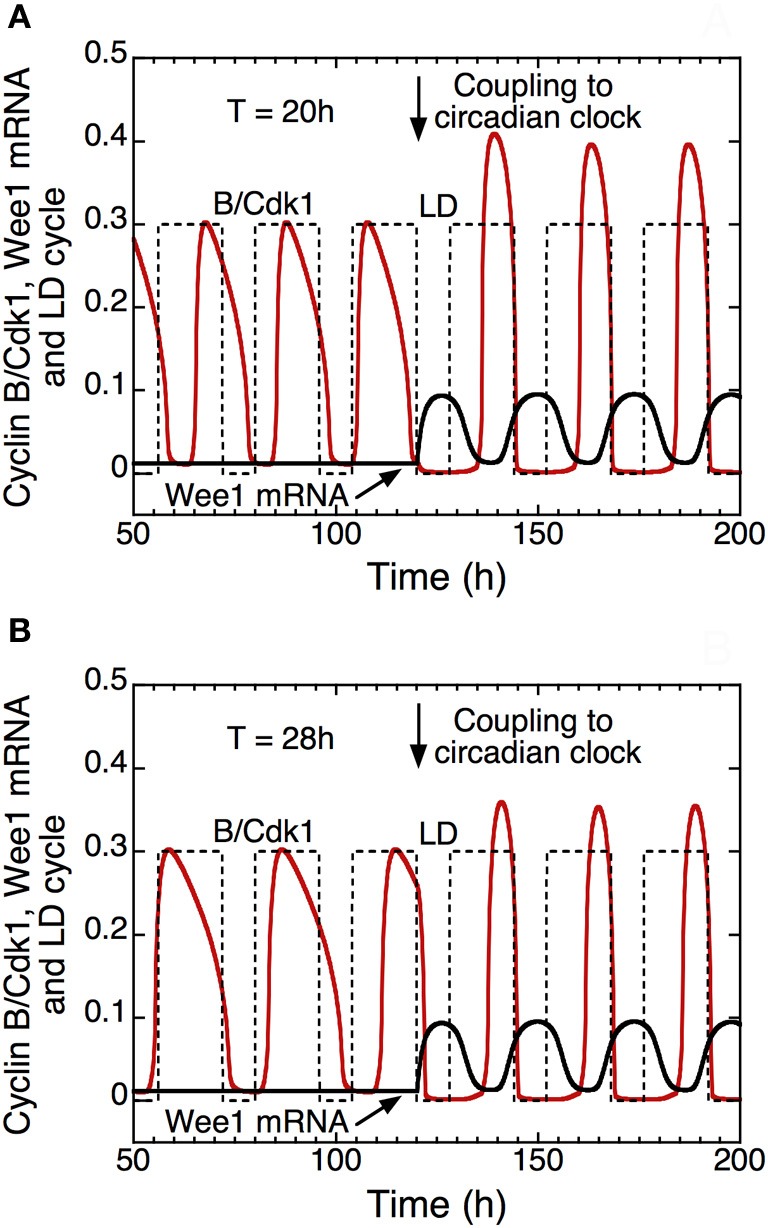
**Entrainment of the mammalian cell cycle by the circadian clock (Gérard and Goldbeter, 2012a).** The curves show the time evolution of cyclin B/Cdk1 (in red), total Wee1 mRNA (in black) and the light/dark (LD) cycle (dashed line) in the absence (*t* < 120 h) or in the presence (*t* > 120 h) of coupling between the detailed model for the Cdk network and the model for the circadian clock in mammals (Leloup and Goldbeter, 2003). From *t* = 120 h on, the rate of synthesis of Wee1 mRNA, *v*_*sw*_, that depends on the circadian complex protein CLOCK/BMAL1, passes from 0 to 0.1 μM.h^−1^. Starting from an autonomous cell cycle period of 20 h in **(A)** or 28 h in **(B)**, the coupling through Wee1 that starts in *t* = 120 h elicits entrainment of the cell cycle period to a period of 24 h. In the model, the LD cycle is defined by an increase of the rate of transcription of the *Per* gene during the light (L) phase (see Leloup and Goldbeter, 2003; Gérard and Goldbeter, 2012a for further details). Upon entrainment, the peak of activity of cyclin B/Cdk1 occurs at the end of the L phase, which corresponds to the experimental observations (Bjarnason et al., 1999). Parameter values are the same as in Figure 3 in Gérard and Goldbeter (2012a).

In the presence of coupling to the circadian clock (see Figure 11, for *t* > 120 h), the model shows that autonomous periods of the cell cycle smaller, e.g., 20 h in **(A)**, or larger than 24 h, e.g., 28 h in **(B)**, can be entrained to oscillate at a circadian period (Gérard and Goldbeter, 2012a). This result demonstrates that the cell cycle can be brought to oscillate at a circadian period when its autonomous period prior to coupling is in an appropriate range. The model indicates that the combination of multiple modes of coupling does not necessarily facilitate entrainment of the cell cycle by the circadian clock. Furthermore, outside the range of entrainment, the coupling to the circadian clock may lead either to disconnected oscillations in the cell cycle and the circadian system, or to complex oscillatory dynamics of the cell cycle in the form of endoreplication, complex periodic oscillations, or even chaos (Gérard and Goldbeter, 2012a).

Previous studies have established the existence of a link between dysregulation of circadian rhythms and cancer (Filipski et al., 2002; Gauger and Sancar, 2005; Sahar and Sassone-Corsi, 2009). The existence of robust circadian rhythms is positively correlated with the health status of cancer patients (Innominato et al., 2009). Entrainment of the cell cycle by the circadian clock could thus play a crucial role in normal cell proliferation and might be deregulated in cancer cells (Fu and Lee, 2003; Pendergast et al., 2010).

### A skeleton model for the mammalian cell cycle

#### Building a skeleton model for the Cdk network

The question arises as to whether the complexity of the detailed model for the Cdk network can be reduced without losing the insights provided by this model. We have shown that most of the results on the oscillatory dynamics can indeed be retained when the number of variables and parameters are significantly reduced in a skeleton model for the Cdk network. A necessary price must be paid for this reduction in complexity. We have to abandon a large number of biochemical details while trying to retain the logic of the regulatory wiring of the Cdk network. The advantage of reducing the number of variables is that the system becomes more amenable to a thorough numerical analysis of its dynamical properties, both in its deterministic and stochastic versions.

A first version of the skeleton network (Gérard and Goldbeter, 2011) containing only five variables did not incorporate Cdk regulation through phosphorylation-dephosphorylation. Without increasing the number of variables we recently extended this skeleton model by incorporating the control of Cdk2 and Cdk1 by phosphatases Cdc25 and kinase Wee1. This inclusion allows us to examine the effect of PF loops exerted on Cdk2 via its activation of phosphatase Cdc25 and on Cdk1 via its activation of Cdc25 and inhibition of kinase Wee1 (Gérard et al., 2012).

**Figure 12:**
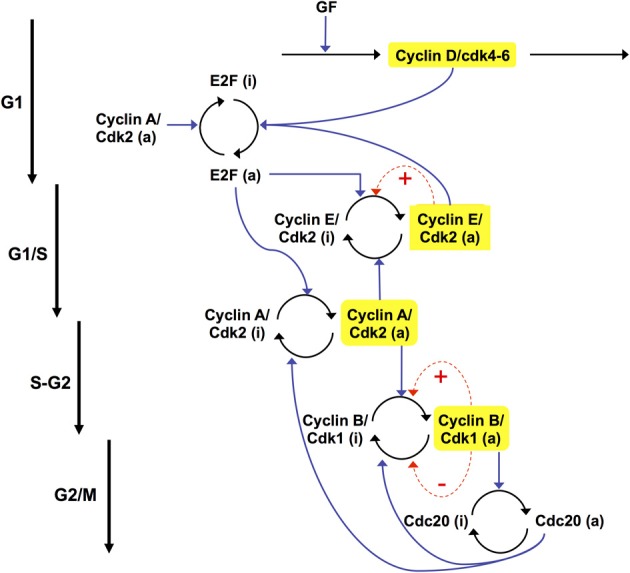
**Scheme of the extended skeleton model for the mammalian cell cycle (Gérard et al., 2012).** The model contains the four main cyclin/Cdk complexes, the transcription factor E2F, and the protein Cdc20. Growth factor (GF) induces entry in the G1 phase of the cell cycle by promoting the synthesis of cyclin D/Cdk4–6. The latter complex allows the activation of E2F, which elicits the synthesis of cyclin E/Cdk2, at the G1/S transition, and cyclin A/Cdk2, during the S phase of DNA replication. During G2, cyclin A/Cdk2 also activates the synthesis of cyclin B/Cdk1, which elicits the peak of activity of cyclin B/Cdk1 at the G2/M transition. During mitosis, cyclin B/Cdk1 activates by phosphorylation the protein Cdc20. This creates a negative feedback loop in the activity of cyclin A/Cdk2 and cyclin B/Cdk1 by promoting the degradation of these complexes. The regulations exerted by Cdc20 allow the cell to complete mitosis, and to start a new cycle if GF is present in sufficient amount. In this version of the model, we consider that the total concentrations of cyclin E/Cdk2, cyclin A/Cdk2, and cyclin B/Cdk1 remain constant. These complexes can vary between an active (a) and an inactive (i) form. Moreover, positive feedback (PF) loops can be added in the regulation of cyclin E/Cdk2 and cyclin B/Cdk1, which control the G1/S and G2/M transitions (dashed arrows in red). These PF loops are due to the mutual activation between Cdk2, Cdk1, and their phosphatase Cdc25 and to the mutual inhibition between Cdk1 and the kinase Wee1.

**Figure 13:**
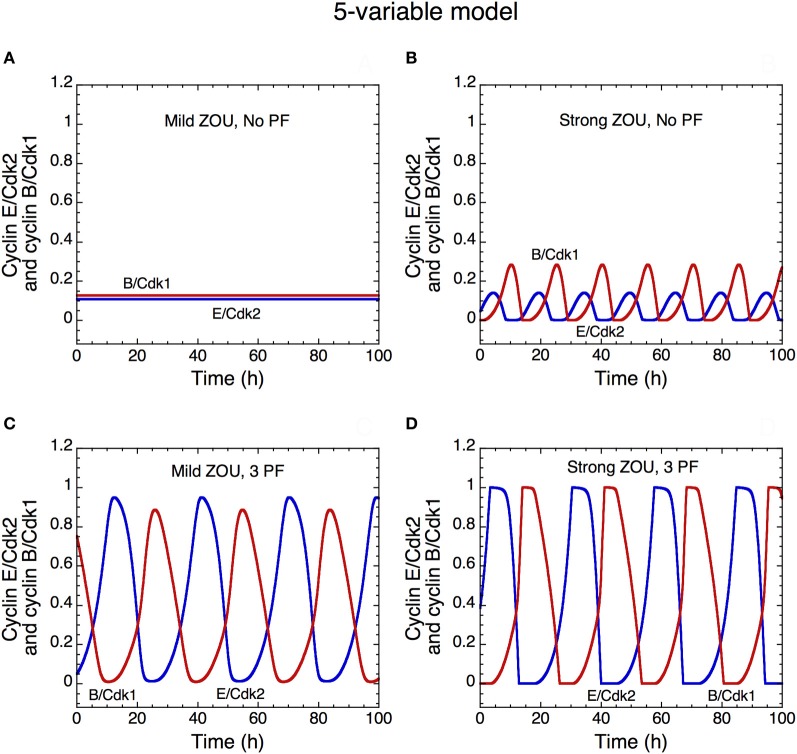
**Oscillatory dynamics of the skeleton model for the Cdk network driving the mammalian cell cycle: role of zero-order ultrasensitivity (ZOU) and positive feedback.** The curves show the time evolution of cyclin E/Cdk2 (in blue) and cyclin B/Cdk1 (in red) in the presence of mild ultrasensitivity (*K* = 0.1 μM in **A** and **C**) or strong ultrasensitivity (*K* = 0.005 μM in **B** and **D**), and in the absence of any PF loop (*b*_1_ = 0, *b*_2_ = 0, *K*_ib_ = 1000 μM in **A**,**B**) or presence (*b*_1_ = 1, *b*_2_ = 1, *K*_ib_ = 0.5 μM in **C**,**D**) of three PF loops. The presence of PF loops as well as the presence of strong ultrasensitivity enhance the amplitude of Cdk oscillations (compare panels **A** and **B** with **C** and **D**). With the value of parameters used in the simulations, the Cdk network tends to a stable steady state in the presence of mild ultrasensitivity and in the absence of any PF loop **(A)**. This result fits with experimental observations showing that bypassing PF loops produces damped oscillations of the cyclin/Cdk complexes (Pomerening et al., 2005). Parameter values are as in Table 2 in Gérard et al. (2012) with *V*_1e2f_ = 0.35 h^−1^, *V*_1Me_ = 1 μM^−1^h^−1^, *V*_1Ma_ = 0.7 h^−1^, *V*_1Mb_ = 1.1 μM^−1^h^−1^, and *V*_1cdc20_ = 4 h^−1^.

The extended skeleton model for the Cdk network is schematized in Figure 12 (see also Gérard et al., 2012). At the beginning of the cell cycle, growth factor GF activates directly the synthesis of the cyclin D/Cdk4–6 complex, which promotes progression in G1. This complex ensures the activation of E2F, which in turn activates the synthesis of cyclin E/Cdk2 and cyclin A/Cdk2. During the G1 phase, cyclin E/Cdk2 reinforces the activation of E2F. During the S phase of DNA replication, cyclin A/Cdk2 activates the degradation of cyclin E/Cdk2, which ensures that the latter complex will not be activated at the end of the cell cycle. Cyclin A/Cdk2 also permits exit of the S phase by allowing the inactivation of E2F and by promoting the synthesis of cyclin B/Cdk1, which brings about the G2/M transition. During mitosis, cyclin B/Cdk1 activates, by phosphorylation, the protein Cdc20. This protein creates two negative feedback loops by promoting the inactivation of cyclin A/Cdk2 and cyclin B/Cdk1, thereby ensuring the completion of the cell cycle. A new cell cycle starts if GF is present in sufficient amounts—see Figure 13 and (Gérard and Goldbeter, 2011) for further details about the regulatory mechanisms of the Cdk network in the skeleton, five-variable, model for the mammalian cell cycle.

#### Oscillations in the skeleton model for the Cdk network: role of ultrasensitivity and positive feedback loops

The skeleton, five-variable, model or the Cdk network is also capable of temporal self-organization in the form of sustained oscillations. As in the detailed model for the cell cycle, sustained oscillations of the various cyclin/Cdk complexes correspond to the evolution toward a limit cycle, which can be reached regardless of initial conditions (Gérard and Goldbeter, 2011; Gérard et al., 2012). Here we focus on the effect of ultrasensitivity and PF loops on the oscillatory dynamics of the Cdk network.

A number of experimental and theoretical studies, mostly devoted to the early cell cycles in amphibian embryos (Goldbeter, 1993; Novak and Tyson, 1993; Ferrell and Machleder, 1998; Pomerening et al., 2003; Sha et al., 2003) and to the yeast cell cycle (Chen et al., 2004) showed that PF contributes to the robustness of oscillatory behavior and make the cell cycle transitions irreversible (Novak et al., 2007). Based on a previous modeling study showing that PF loops increase the robustness of Cdk oscillations toward molecular noise (Gonze and Hafner, 2010; Gérard et al., 2012), we illustrate here the effect of PF loops on the dynamics of the cell cycle.

The time evolution of cyclin E/Cdk2 and cyclin B/Cdk1 in the extended version of the skeleton model is shown in the presence of mild (Figures 13A,C) or strong ultrasensitivity (Figures 13B,D), and in the absence (Figures 13A,B) or presence (Figures 13C,D) of 3 PF loops in the G1/S and G2/M transitions of the cell cycle. The presence of PF loops as well as the degree of ultrasensitivity in phosphorylation–dephosphorylation of Cdk1 and Cdk2 contribute to the robustness of oscillations in the Cdk network by augmenting their amplitude (see Gérard et al., 2012; and also Figure 13). The ultrasensitivity considered here originates from zero-order kinetics for the kinases and phosphatases involved in phosphorylation-dephosphorylation (Goldbeter and Koshland, 1981). The results likely extend to other sources of ultrasensitivity such as multiple phosphorylation or substrate competition (Gunawardena, 2005; Kim and Ferrell, 2007; Barik et al., 2010; Trunnell et al., 2011).

**Figure 14:**
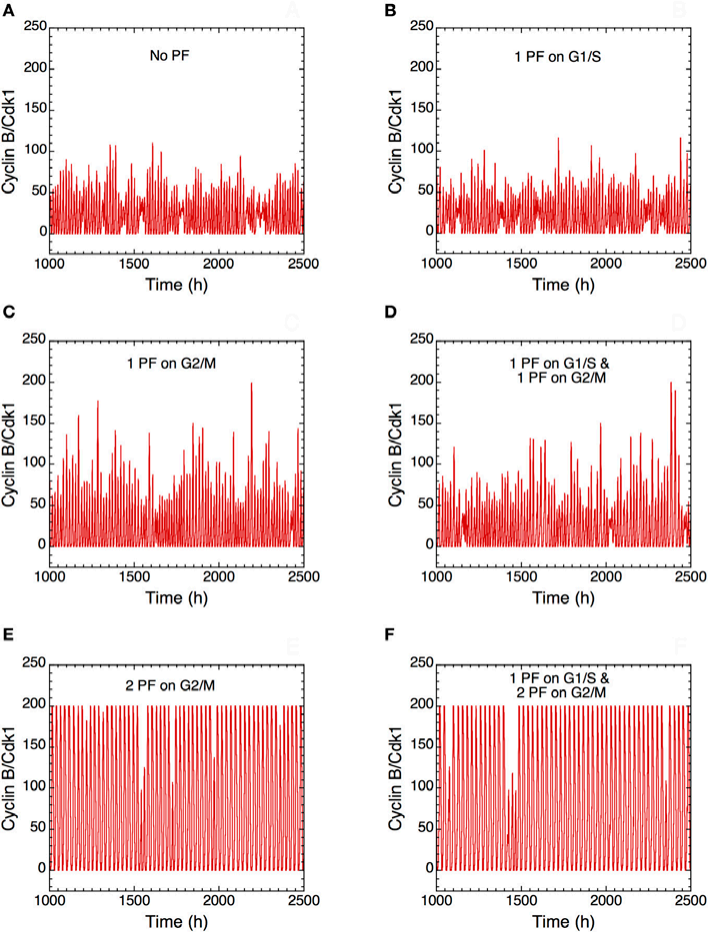
**Positive feedback loops increase the robustness of Cdk oscillations with respect to molecular noise.** The long-term time evolution of cyclin B/Cdk1 is shown in the extended version of the skeleton model for the mammalian cell cycle (Gérard et al., 2012) **(A)** in the absence of PF loop (*b*_1_ = 0, *b*_2_ = 0, *K*_ib_ = 1000 μM) or in the presence of **(B)** one PF on G1/S via Cdc25 (*b*_1_ = 1, *b*_2_ = 0, *K*_ib_ = 1000 μM), **(C)** one PF on G2/M via Cdc25 (*b*_1_ = 0, *b*_2_ = 1, *K*_ib_ = 1000 μM), **(D)** one PF on G1/S and one PF on G2/M via Cdc25 (*b*_1_ = 1, *b*_2_ = 1, *K*_ib_ = 1000 μM), **(E)** two PF on G2/M via Cdc25 and Wee1 (*b*_1_ = 0, *b*_2_ = 1, *K*_ib_ = 0.5 μM), and **(F)** one PF on G1/S and two PF on G2/M (*b*_1_ = 1, *b*_2_ = 1, *K*_ib_ = 0.5 μM). All numerical simulations were performed in the presence of strong ultrasensitivity (*K* = 0.005 μM). The combination of two PF on G2/M markedly enhances the robustness of Cdk oscillations toward molecular noise (compare panels **E** and **F** with panels **A–D**). Parameter values are as in Tables 2 and 3 in Gérard et al. (2012) with *V*_1e2f_ = 0.35 h^−1^, *V*_1Me_ = 1 μM^−1^h^−1^, *V*_1Ma_ = 0.7 h^−1^, *V*_1Mb_ = 1.1 μM^−1^h^−1^, and *V*_1cdc20_ = 4 h^−1^.

**Figure 15:**
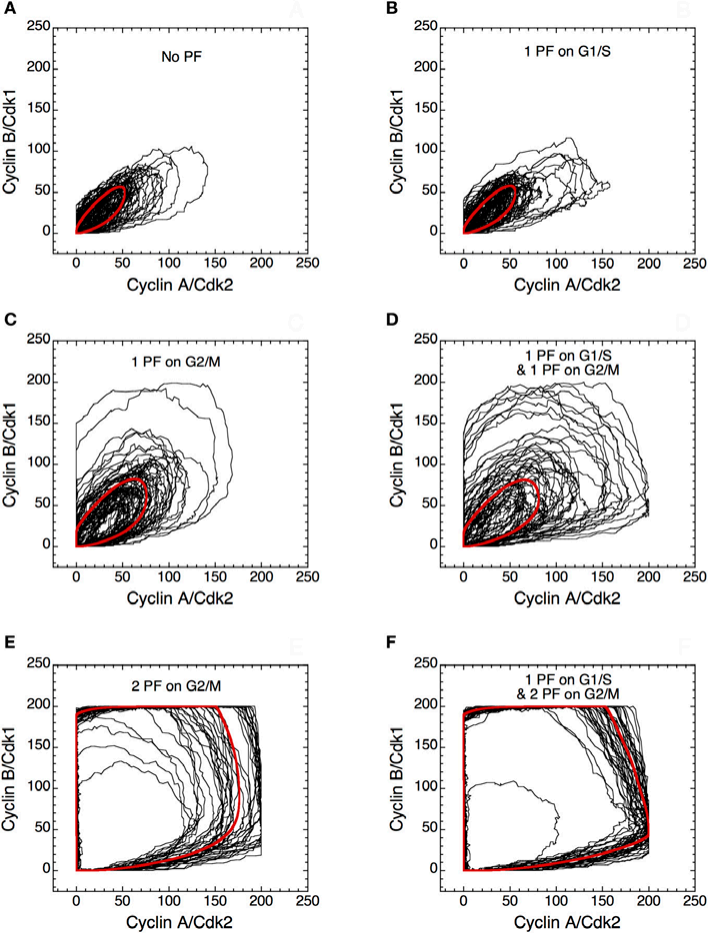
**Positive feedback loops increase the robustness of limit cycle oscillations in the Cdk network.** Projection of the stochastic limit cycle (black curve) and the corresponding deterministic limit cycle (red curve) in the cyclin A/Cdk2 *vs.* cyclin B/Cdk1 plane. Numerical simulations were performed in the extended version of the skeleton model for the mammalian cell cycle (Gérard et al., 2012) **(A)** in the absence of PF loop (*b*_1_ = 0, *b*_2_ = 0, *K*_ib_ = 1000 μM), or in the presence of **(B)** one PF on G1/S via Cdc25 (*b*_1_ = 1, *b*_2_ = 0, *K*_ib_ = 1000 μM), **(C)** one PF on G2/M via Cdc25 (*b*_1_ = 0, *b*_2_ = 1, *K*_ib_ = 1000 μM), **(D)** one PF on G1/S and one PF on G2/M via Cdc25 (*b*_1_ = 1, *b*_2_ = 1, *K*_ib_ = 1000 μM), **(E)** two PF on G2/M via Cdc25 and Wee1 (*b*_1_ = 0, *b*_2_ = 1, *K*_ib_ = 0.5 μM), and **(F)** one PF on G1/S and two PF on G2/M (*b*_1_ = 1, *b*_2_ = 1, *K*_ib_ = 0.5 μM). All simulations were performed in the presence of strong ultrasensitivity (*K* = 0.005 μM). Parameter values are as in Tables 2 and 3 in Gérard et al. (2012), with *V*_1e2f_ = 0.35 h^−1^, *V*_1Me_ = 1 μM^−1^ h^−1^, *V*_1Ma_ = 0.7 h^−1^, *V*_1Mb_ = 1.1 μM^−1^ h^−1^, and *V*_1cdc20_ = 4 h^−1^.

The presence of PF loops also increases the robustness of Cdk oscillations toward molecular noise (Gérard et al., 2012). The skeleton model is more amenable to stochastic simulations than the detailed version of the model for the Cdk network. The results of such stochastic simulations are shown in Figures 14 and 15. The time evolution of cyclin B/Cdk1 as well as the projection, in the cyclin A/Cdk2 *vs.* cyclin B/Cdk1 plane, of the stochastic limit cycle (black curve) together with the corresponding deterministic limit cycle (red curve) are shown in Figures 14 and 15, respectively, in the absence of PF **(A),** in the presence of 1 PF on G1/S via Cdc25 **(B),** 1 PF on G2/M via Cdc25 **(C)**, 1 PF on G1/S and 1 PF on G2/M via Cdc25 **(D)**, 2 PF on G2/M via Cdc25 and Wee1 **(E)**, or 3 PF, i.e., 1 PF on G1/S via Cdc25 and 2 on G2/M via Cdc25 and Wee1 **(F).** To display many cycles the time series in Figure 14 are obtained for extended durations, so as to better illustrate the degree of irregularity of the oscillations in the presence of noise; shorter stochastic time series in which details of changes in Cdk1 activity are more visible can be found elsewhere (Gérard et al., 2012). The results of Figures 14 and 15 indicate that the presence of multiple PF loops reinforces the robustness of Cdk oscillations toward molecular noise. Moreover, the model predicts that the redundant PF loops on the G2/M transition through Cdc25 and Wee1 have a synergistic effect on the robustness of the cell cycle toward molecular noise (compare panels E, F with panels A–D in Figures 14 and 15).

## Discussion

The use of computational models is justified in Systems Biology by the complexity of cellular networks in which large numbers of variables are coupled through multiple regulatory interactions. When addressing by means of computational modeling the dynamics of the Cdk network that drives the mammalian cell cycle, we first have to deal with the very complexity of this key cellular regulatory system: what level of biochemical detail is most appropriate for grasping the dynamics of this key cellular network? Here we showed that it is possible to address this key issue in several, complementary ways. We first built a rather detailed model for the Cdk network that allows a thorough analysis of its dynamic behavior in physiological and pathological conditions. In a second stage we indicated how to reduce the complexity of this detailed model by considering a skeleton version in which many biochemical details are relinquished. The skeleton model, based on the same regulatory backbone, retains most dynamical properties of the detailed one.

The two versions of the model for the Cdk network schematized in Figures 1 and 12, respectively, differ markedly by the number of variables: 39 in the detailed model (Gérard and Goldbeter, 2009) versus only five in the skeleton version (Gérard and Goldbeter, 2011; Gérard et al., 2012). Both versions of the model show that the network can self-organize in time in the form of sustained oscillations of the various cyclin/Cdk complexes, which allow for an ordered progression along the different phases of the mammalian cell cycle (see Figure 2 and also Gérard and Goldbeter, 2009, 2011; Gérard et al., 2012). The main result of this computational approach is to associate the transition from cellular quiescence to cell proliferation with the switch from a stable steady state to sustained oscillations in the Cdk network. The latter drives a cell cycle clock, which belongs to the class of cellular rhythms, alongside other genetic and biochemical oscillators, the list of which continues to increase, which range from glycolytic oscillations in yeast and pancreatic β cells to cyclic AMP oscillations in *Dictyostelium* amoebae, and from oscillations in p53 and NF-κB to circadian rhythms and the segmentation clock (Goldbeter, 1996; Goldbeter et al., 2012).

The 39-variable model already represents a reduced version of a full model for the Cdk network, because enzymatic reactions are assumed to obey Michaelis–Menten kinetics. This assumption is not necessarily valid, because substrates of kinases and phosphatases, such as Cdks, are themselves kinases and are therefore not necessarily in excess with respect to the enzymes that catalyze their covalent modification. In such a case, one can either use an extended form of the Michaelis–Menten equation (Segel, 1988; Ciliberto et al., 2007) or one can resort to descriptions in terms of mass-action kinetics. When adopting the latter treatment for all enzymatic steps, enzyme-substrate complexes become themselves variables so that the number of the latter increases from 39 up to some 80 variables. The numerical analysis of this more complex model shows that it retains the oscillatory properties of the 39-variable version, which therefore represents a good approximation for studying the oscillatory behavior of the Cdk network (Gérard and Goldbeter, 2012b).

Because it incorporates the main actors of the cell cycle control machinery, the 39-variable model is particularly useful to study the behavior of the Cdk network in the presence of overexpression or deletion of a variety of factors that impinge on the dynamics of the cell cycle. The model indicates that a balance between proteins that promote (oncogenes) or hinder (tumor suppressors) governs progression in the cell cycle. The role of this balance is well illustrated by the antagonistic effects of pRB, which inhibits, and E2F, which promotes, progression in the cell cycle (Figure 3). The model shows that, depending on the relative levels of E2F and pRB, cell proliferation can become independent of the presence of growth factor. Such independence is often considered as a hallmark of cancer cells, many of which do not require the presence of growth factor to proliferate, when they do not secrete themselves their own growth factor (Hanahan and Weinberg, 2000).

The computational approach provides a true systemic view of the dynamics of the Cdk network that drives the mammalian cell cycle. Thus, it shows that there are multiple ways to trigger the transition from cellular quiescence to cell proliferation. As illustrated in Figure 4, this transition could be achieved by overexpressing oncogenes or transcription factors, or by repressing tumor suppressors or Cdk inhibitors. Any permanent change that tilts the balance from quiescence to proliferation will trigger the transition from steady state to sustained Cdk oscillations. The resulting abusive progression in the cell cycle might correspond to the dynamical behavior of cancer cells (Hanahan and Weinberg, 2000).

The behavior of the detailed model for the Cdk network can be compared with a large number of experimental observations on the mammalian cell cycle. Such a comparison bears on the existence of a restriction point in G1 beyond which the cell does not need the presence of growth factor to complete a cycle (Gérard and Goldbeter, 2009). The model can also reproduce the observation that the cell cycle can proceed in the sole presence of Cdk1 (Santamaria et al., 2007). The model for the Cdk network shows that oscillations can indeed occur in the presence of only Cdk1, provided that the latter can form complexes with the different cyclins D, E, A, and B (Gérard and Goldbeter, 2009).

The computational approach to the cell cycle further allows us to study the transition between cellular proliferation and cell differentiation, which begins by arresting the cell cycle in G1. Such an arrest in the G1 phase can be achieved by overexpressing p21/p27 or pRB, as shown in Figure 5. This cell cycle arrest might correspond to a prerequisite to cell differentiation (Evers, 1999; Quaroni et al., 2000; Ilyin et al., 2003). Indeed, the dynamical switch between proliferation and differentiation is often initiated through signaling that leads to an increase in p21/p27 (Hara et al., 2011).

The dynamics of the cell cycle is controlled by the presence of multiple checkpoints, which ensure correct progression in the cell cycle (Hartwell and Weinert, 1989). The question arises as to how checkpoints affect the oscillatory dynamics of the Cdk network. To address this issue we incorporated into the detailed model for the Cdk network the DNA replication checkpoint controlled by the kinases ATR and Chk1. Numerical simulations indicate that progression in the cell cycle slows down when the activation of the checkpoint increases. Moreover, the presence of the checkpoint results in better separation between DNA replication and mitosis (see Figures 8 and 9). The dynamics of the Cdk network remains, however, fundamentally unaltered: beyond the changes in the waveform of periodic changes in Cdk activity, the temporal behavior of the network retains its oscillatory nature. If, however, the activation of the checkpoint becomes too strong, progression in the cell cycle may be arrested.

In analyzing the mechanism of Cdk oscillations in the detailed model for the mammalian cell cycle, we used bifurcation diagrams to show that the dynamical behavior of the Cdk network rests on a cascade of bistable switches in the activation of the various cyclin/Cdk complexes that govern progression in G1, S, G2, and M (see Figure 10). Such a bifurcation analysis has previously been used for studying the dynamics of the yeast and mammalian cell cycles, considering cell mass as bifurcation parameter (Novak and Tyson, 1993, 2004; Chen et al., 2004). Here, the bifurcation diagrams indicate that the role of the first three modules of the Cdk network is to progressively build up the level of cyclin A/Cdk2. When the latter exceeds a threshold, the last module of the network enters a domain of sustained oscillations: a first pulse in cyclin B/Cdk1 occurs—this is the first peak starting the oscillations in Cdk1. These oscillations would be sustained if the level of cyclin A/Cdk2 were maintained at a constant level. However, the pulsatile increase in cyclin B/Cdk1 activates APC/Cdc20, leading to the degradation of cyclin A (and later, cyclin B) followed by a decrease in cyclin A/Cdk2. As a consequence of this drop in cyclin A/Cdk2, cyclin B/Cdk1 leaves the domain of sustained oscillations. The drop in cyclin A/Cdk2 and cyclin B/Cdk1 resets the Cdk network to the conditions where the four modules are inactivated; the cell cycle starts again in the G1 phase when the first module of the network is activated by the synthesis of cyclin D due to the presence of GF. The presence of a negative feedback loop between cyclin B/Cdk1 and APC/Cdc20 at the end of the network allows the cell to enter transiently into a domain of sustained oscillations of Cdk1, which turns the whole Cdk network into a limit cycle oscillator (Figures 10C,D).

The detailed model for the Cdk network was also used to study entrainment of the cell cycle by the circadian clock through circadian control of the kinase Wee1 (see Figure 11 and also Gérard and Goldbeter, 2012a). Additional coupling of the cell cycle to the circadian clock also occurs through cell cycle factors such as p21 or cyclin E. The domain of entrainment does not increase, however, when the cell cycle is coupled to the circadian clock through multiple links (Gérard and Goldbeter, 2012a). The primary reason for the multiplicity of links between the cell cycle and the circadian clock may thus be to ensure redundancy in coupling. Circadian entrainment may prove important for correct operation of the cell cycle in healhy cells (Pendergast et al., 2010).

In building a skeleton version of the Cdk network we aimed at reducing the number of variables without losing the backbone of its regulatory structure. Thus, we abandoned many variables, such as pRB or Cdh1, and biochemical details, but kept the essential ingredient of the regulatory wiring: each Cdk module promotes the activation of subsequent Cdk modules and inhibits the previous modules in the network (Gérard and Goldbeter, 2011; Gérard et al., 2012). That the skeleton model produces the same kind of oscillations as the detailed model corroborates the view that the oscillatory dynamics represents a structurally stable mode of operation of the Cdk network. In view of its complexity, it is not surprising that the Cdk regulatory network contains several oscillatory circuits, resulting from the presence of multiple negative feedback loops involving Cdk2 and/or Cdk1 (see Figure 6). These multiple circuits are embedded in both the detailed and skeleton versions of the model for the Cdk network (Gérard and Goldbeter, 2009, 2011). Because the oscillatory circuits are tightly coupled in the Cdk network, they synchronize so that the latter generally undergoes simple periodic oscillations of the limit cycle type. When the coupling strength between the oscillatory circuits diminishes, these circuits are able to express their own oscillatory potential, so that more complex patterns of oscillations may develop. Such patterns include endoreplication cycles or tetraploidy (see Figure 7), as well as quasi-periodic oscillations and even chaos (Gérard and Goldbeter, 2010).

Numerous studies have emphasized the role of PF loops and bistability in the dynamics of the cell cycle. This aspect has been addressed both in experiments and in models for the early cell cycles in amphibian embryos (Goldbeter, 1993; Novak and Tyson, 1993; Ferrell and Machleder, 1998; Pomerening et al., 2003, 2005; Sha et al., 2003) and for the yeast cell cycle (Dirick and Nasmyth, 1991; Chen et al., 2004; Domingo-Sananes et al., 2011). The role of PF loops leading to bistability was also stressed experimentally and in partial models for the mammalian cell cycle (Hoffmann et al., 1994; Skotheim et al., 2008; Yao et al., 2008; Ferrell et al., 2009).

Due to its relative simplicity and smaller numbers of variables and parameters, the skeleton model for the Cdk network is particularly helpful for studying the effect of PF loops on the dynamics of the cell cycle (Gérard et al., 2012). The model shows that the presence of PF in the regulation of Cdk activity as well as ultrasensitivity in the phosphorylation/dephosphorylation mechanisms (Goldbeter and Koshland, 1981) both increase the amplitude of oscillations of the various cyclin/Cdk complexes (see Figure 13 and Gérard et al., 2012). Stochastic simulations of the skeleton model further show that redundant PF loops, especially in the G2/M transition of the cell cycle, markedly enhance the robustness of oscillations toward molecular noise (see Figures 14, 15 and Gérard et al., 2012).

Detailed models for the Cdk network can help to highlight the differences that might arise from a dynamical point of view between the cell cycle in healthy and cancer cells as a result of overexpression of oncogenes and/or deletion of tumor suppressors (Gérard and Goldbeter, 2009; Tyson et al., 2011). On the other hand the skeleton model for the cell cycle (Gérard and Goldbeter, 2011; Gérard et al., 2012) provides a good tool to study the effect of regulatory motifs, such as PF loops, on the dynamics of the mammalian cell cycle. Such models might also prove useful to study the dynamics of desynchronization in a cell population.

Models of increasing complexity have been discussed here for the Cdk network driving the mammalian cell cycle. They range from a detailed, 39-variable model to a skeleton model containing only five variables. Much as an even more complex version of the detailed model, which counts no less than 80 variables (Gérard and Goldbeter, 2012b), all these models show that the Cdk network is capable of temporal self-organization in the form of sustained Cdk oscillations. The onset of oscillatory behavior around an unstable steady state corresponds to the passage from cellular quiescence to cell proliferation. Similar oscillations were observed in a model, containing only three variables, proposed for the amphibian embryonic cell cycle (Goldbeter, 1991); the latter model is based on the negative feedback exerted on Cdc2, the analog of Cdk1, via its activation of cyclin B degradation. Regardless of their degree of complexity, all these models generate limit cycle oscillations and therefore support the view that the cell cycle is a true cellular clock.

### Conflict of interest statement

The authors declare that the research was conducted in the absence of any commercial or financial relationships that could be construed as a potential conflict of interest.

## Research Topic:

From structural to molecular systems biology: experimental and computational approaches to unravel mechanisms of kinase activity regulation in cancer and neurodegeneration

## Topic Editor:

Matteo Barberis, Humboldt University Berlin, Germany; Max Planck Institute for Molecular Genetics, Berlin, Germany
